# Shifts in soil microbiome surrounding a thermal treatment facility for hazardous waste: the hidden impact of environmentally persistent free radicals

**DOI:** 10.1039/d5em00439j

**Published:** 2026-04-27

**Authors:** Fan Zhang, Myron L. Lard, Lavrent Khachatryan, Chuqi Guo, Aiyanah Sandifer, Nora M. Villafuerte, Divine B. Nde, Robert L. Cook, Stephania A. Cormier, Jennifer Richmond-Bryant

**Affiliations:** a Department of Biological Sciences, Louisiana State University Baton Rouge LA 70803 USA fanzhang@lsu.edu; b Department of Chemistry, Louisiana State University Baton Rouge LA 70803 USA; c Department of Biological Sciences, Louisiana State University and Pennington Biomedical Research Center Baton Rouge LA 70803 USA; d Department of Forestry and Environmental Resources, North Carolina State University Raleigh NC 27695 USA

## Abstract

The disposal of hazardous materials from Superfund sites often involves thermal treatment (TT), generating environmentally persistent free radicals (EPFRs). While substantial evidence links EPFR exposure to negative health outcomes, its effects on the soil microbiome remain underexplored. Since the mid-1980s, a TT facility in Colfax, LA, has employed open-burn and open-detonation to process hazardous waste. In 2023, we collected soil samples from 13 residential sites within a 17-km radius of the TT facility and analyzed microbial communities and EPFR content. Our findings revealed a distinct microbial community near the TT facility (within 5-km), characterized by reduced bacterial abundance and increased fungal presence. Soil EPFR concentrations ranged from 0.81 × 10^16^–4.39 × 10^16^ spins per g with *g*-factor values of 2.0033–2.0040, indicating a mixture of carbon-centered radicals with adjacent oxygen and oxygen-centered radicals. Correlation analysis identified bacterial taxa, particularly *Alpha-proteobacteria* and *Actinobacteria*, positively associated with EPFR abundance. *In vitro* tests showed that laboratory generated EPFRs more strongly inhibited bacterial growth than fungal growth, though some bacterial isolates from the study sites exhibited resistance to EPFR exposure. The differences in microbial responses to EPFR exposure may contribute to the shifts in microbial communities near the TT facility. Our study advances the understanding of EPFR impacts on the soil microbiome and suggests potential long-term effects on environmental and community health.

## Abstract 2

Changes in soil microbiome near a thermal treatment facility, together with spatial variation in EPFR *g*-factor values, indicate higher oxygen-centered radicals near the source. Microcosm experiments show radicals differentially affect soil microbes.

## Graphical abstract

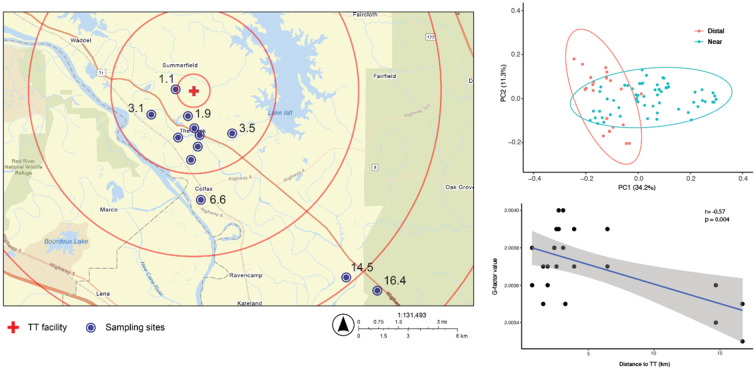

**Environmental significance**

Our study examined changes in microbial abundance and composition in relation to Environmentally Persistent Free Radicals (EPFRs) near a hazardous waste thermal treatment facility. With laboratory-generated EPFR exposures, we further explored their potential role in driving microbial community changes. To our knowledge, this is the first study to link EPFR exposure with soil microbiome alterations in a natural ecosystem. We propose oxidative stress as a key molecular mechanism underlying these shifts. While EPFRs are recognized for their direct toxicity, our findings highlight their potential to indirectly affect soil health by reshaping microbial communities critical to nutrient cycling, ecosystem resilience, and public health.

## Introduction

Environmentally persistent free radicals (EPFRs) originate from electron-rich aromatic hydrocarbons that react with metals, forming free radicals on the surface of substrate catalysts like silica.^1–5^ These highly reactive particles can persist in the environment for extended periods, ranging from weeks to years.^6,7^ Studies have shown that EPFRs can induce oxidative stress and cause cellular damage in animals or humans, leading to cardiopulmonary dysfunction and other health issues.^5,8–14^ Thermal treatment (TT) facilities for hazardous waste are a significant source of anthropogenic EPFRs. During thermal remediation processes, organic compounds and transition metals in the waste are converted into EPFRs, along with their precursor polycyclic aromatic hydrocarbons (PAHs), which are then released into the environment.^3,4^ These airborne pollutants can infiltrate into local soil environment through deposition,^1,2^ where interactions with complex soil chemicals, including metals and organics, enable the accumulation and continuous generation of EPFRs.^15^

In addition to their health effects, EPFRs have been shown to adversely affect soil bacteria growth, community diversity, and the health of soil fauna, such as earthworms, causing tissue damage and gut dysbiosis.^16–18^ However, the long-term impact of EPFRs on the soil microbiome, particularly on both bacterial and fungal communities, has yet to be fully assessed and experimental evidence comparing their responses to EPFR exposure is still limited. We hypothesize that EPFR exposure could potentially alter the composition and functions of the soil microbial community through both direct and indirect mechanisms. Directly, EPFRs generate reactive oxygen species (ROS), leading to excessive oxidative stress that damages cellular components and organelles.^9,12^ While microbes can counteract this stress using enzymes like superoxide dismutase, catalase, and peroxidases, as well as small molecules like glutathione and thioredoxin that neutralize ROS, the external stress environment can still alter microbial metabolism and impair viability.^16,19^ Indirectly, EPFRs can interact with soil organic matter and minerals, altering the soil's nutrient availability and pH, which in turn drive shifts in the microbial community.^18,20,21^

As a first step, to test this hypothesis, we conducted a field study on topsoil samples collected from residential areas surrounding a TT facility in Colfax, Louisiana. The facility, operational since the mid-1980s, has processed hazardous materials, including munitions, fireworks, and soil from Superfund sites using open-burn and open-detonation (OBOD) methods. At its peak of operations in 2012, the facility burned or detonated 499 515 pounds in net explosive waste, with an estimated gross weight of 830 494–1 373 666 pounds for that year. After that peak, operations fell linearly until OBOD ceased at the end of 2023 [Fig. S1]. Deposition rates for EPFRs for the Colfax sites in total PM have been reported by passive sampling data.^22^ Total EPFRs deposition rates were highest for Spring, 2022 for four out of five sites, with EPFR deposition rate at sites 1.2 km to the west and 2.1 km directly south of the facility more than twice the deposition rates for the other three quarters when sampling took place, reaching a maximum of 1.78 × 10^14^ radicals per dm^2^ per day. These results indicated substantial spatial and temporal variability in EPFR deposition. Pollutant concentration heat maps in Colfax correlate with local health issues, including thyroid disease, skin damage, respiratory problems, and cancer.^23^ In this study, we quantified the abundance of bacteria, fungi, and EPFRs in the soil samples, profiled the composition of bacterial and fungal communities using phylogenetic marker genes, and performed correlation analyses to explore associations between field measurements. Additionally, we conducted microcosm experiments with laboratory-generated EPFRs to validate field observation and identify microbial isolates that may exhibit adaptation to EPFR exposure.

## Material and methods

### Sampling sites

**Fig. 1:**
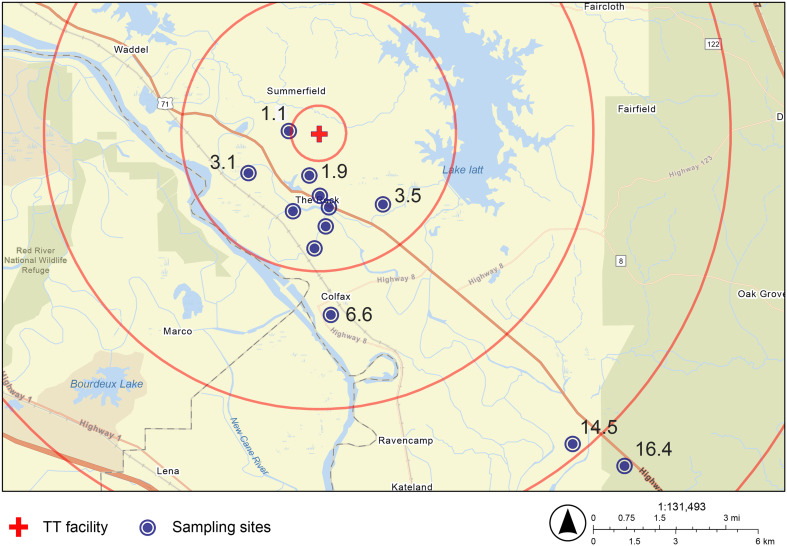
TT facility and sampling sites in Colfax, LA. Numbers represent the distance (km) of each sampling site to the TT facility. The innermost red circle indicates a 1 km radius, followed by concentric rings at 5 km, 10 km, and 15 km.

In 2023, a total of 83 topsoil samples were collected from 13 sites in Colfax, LA (Table 1) to assess the soil microbiome and EPFR concentrations. Sampling was carried out during the spring and summer, corresponding to periods of peak burning activity at the TT facility and recorded hazardous levels of airborne PM_2.5_ particles. Soil sampling locations were selected based on a combination of meteorological data and community-informed survey to identify areas with the highest potential for deposition from the TT facility. Historical wind data [Fig. S2] were evaluated to characterize prevailing wind patterns and atmospheric dispersion conditions during the study period. Wind rose analysis indicated a substantial proportion of calm conditions, as well as seasonally variable wind directions, suggesting the potential for multidirectional dispersion of emissions from the OBOD operations rather than transport along a single dominant plume axis. Therefore, sampling sites were distributed across residential areas within the zone of potential impact rather than restricted to a single downwind transect. In addition, community engagement informed site selection. Interviews with local residents and an on-site community tour conducted prior to soil sampling identified residential areas of concern, which were primarily located south of the facility. Very few residences were located within 10 km north of the facility and none within 5 km to the north. Accordingly, sampling efforts prioritized accessible residential properties most likely to experience potential deposition while maintaining spatial coverage across increasing distance from the facility. Ten sampling locations located within a 5 km radius of the TT facility were designated as “near sites”, while three sites located between 5–15 km served as “distal sites” for comparative analysis [Fig. 1]. Soil samples were collected using stainless steel trowels for surface samples (1–3 cm depth) and an auger for deeper samples (5–10 cm depth). Approximately 20 g of soil were collected from representative locations within the study area, transferred using a sterilized spatula into Ziploc bags for bacterial isolation and microbial abundance quantification, and into sterilized 15 mL tubes prefilled with 4 mL of DNA stabilizing solution for genomic DNA extraction. After collecting each sample, the trowels were cleaned with alcohol wipes.^24^ Three replicates were collected from each site.

**Table 1:** Overview of 13 study sites in Colfax, LA. The number of top soil samples collected at each site are listed under the study site column. Distance refers to the distance from each sampling site to the TT facility

Study sites	Category	Distance (km)	Season	EPFR abundance (average spin per g)	EPFR characteristics (g values)
01 (*n* = 4)	Distal	16.4	Summer	2.48 × 10^16^	2.0034/carbon-centered radicals with adjacent oxygen
02 (*n* = 8)	Distal	14.5	Spring, summer	2.61 × 10^16^	2.0035/carbon-centered radicals with adjacent oxygen
03 (*n* = 11)	Distal	6.6	Spring, summer	2.05 × 10^16^	2.0038/predominantly oxygen-centered radicals
04 (*n* = 4)	Near	4.2	Summer	3.27 × 10^16^	2.0038/predominantly oxygen-centered radicals
05 (*n* = 9)	Near	4.0	Spring, summer	n.a.	n.a.
06 (*n* = 2)	Near	3.5	Summer	1.54 × 10^16^	2.0035/carbon-centered radicals with adjacent oxygen
07 (*n* = 4)	Near	3.4	Summer	2.32 × 10^16^	2.0039/predominantly oxygen-centered radicals
08 (*n* = 4)	Near	3.1	Summer	2.55 × 10^16^	2.0040/predominantly oxygen-centered radicals
09 (*n* = 6)	Near	2.9	Summer	1.18 × 10^16^	2.0038/predominantly oxygen-centered radicals
10 (*n* = 4)	Near	2.7	Summer	2.34 × 10^16^	2.0038/predominantly oxygen-centered radicals
11 (*n* = 4)	Near	2.3	Summer	1.26 × 10^16^	2.0037/predominantly oxygen-centered radicals
12 (*n* = 12)	Near	1.9	Spring, summer	2.90 × 10^16^	2.0036/carbon-centered radicals with adjacent oxygen
13 (*n* = 11)	Near	1.1	Spring, summer	2.11 × 10^16^	2.0037/predominantly oxygen-centered radicals

### Quantification of bacterial and fungal abundance

1 g of soil from each sample was weighed and preserved with 10 mL of 4% formaldehyde in 1X PBS and stored at 4 °C. Samples were vortexed, and 1 mL aliquots were removed before particles could sediment and added to 9 mL of 1X PBS to create an initial suspension. This mixture was vortexed for 2 minutes to disperse the microbes. A series of ten-fold serial dilutions (10^−1^ to 10^−6^) were prepared by transferring 1 mL from the initial suspension to subsequent tubes containing 9 mL of 1X PBS. Next, 1 mL of the final dilution was stained with a 1 : 5000 dilution of SYBR Green I (Invitrogen S7567) for 15 minutes. Bacteria and fungi were collected by vacuum filtration onto a 25 mm Anodisc inorganic filter membrane with a 0.2 µm pore size (Whatman WHA68096022). Cells were enumerated under blue excitation (FITC filter set) using an epi-fluorescent microscope (Nikon Ti_2_ Eclipse).^25^ At least 100 cells from a minimum of 10 fields were counted per filter. The cell abundance per gram of soil was calculated by averaging the number of bacteria or fungi per field, then scaling according to the ratio of the filtered area to the field and the dilution series.

### Genomic DNA extraction, amplicon library construction, and sequencing

Genomic DNA was extracted from soil pellets using the ZymoBIOMICS DNA Kit (Zymo Research) according to the manufacturer's protocol. The quality of the extraction was assessed using a NanoDrop spectrophotometer, ensuring a DNA concentration greater than 10 ng µL^−1^ and high purity, with a 260/280 ratio ranging between 1.8 and 2.0. The extracted DNA was then used as a template for PCR amplification of the V4/5 region of the bacterial 16S rRNA gene (using primers 515F/806R) and the fungal ITS2 gene (using primers ITS3-2024F/ITS4-2409R) to generate barcode-tagged PCR amplicons. All PCR reactions were conducted with 15 µL of Phusion® High-Fidelity PCR Master Mix (New England Biolabs), 2 µM of forward and reverse primers, and approximately 10 ng of template DNA. The PCR cycling conditions included an initial denaturation at 98 °C for 1 minute, followed by 30 cycles of denaturation at 98 °C for 10 seconds, annealing at 50 °C for 30 seconds, and elongation at 72 °C for 30 seconds, with a final extension at 72 °C for 5 minutes. The PCR products were mixed with 1X loading buffer containing SYBR Green and analyzed by electrophoresis on a 2% agarose gel for detection. The PCR products were then pooled in equidensity ratios, and the resulting mixture was purified using the Qiagen Gel Extraction Kit (Qiagen, Germany). The quality of the purified library was assessed using a Qubit® 2.0 Fluorometer (Thermo Scientific) and an Agilent Bioanalyzer 2100 system. Finally, the library was sequenced on an Illumina NovaSeq platform, generating 250 bp paired-end reads.

### Analysis of microbiome composition

Paired-end reads were assigned to samples based on their unique barcodes, and the barcodes and primer sequences were trimmed. The paired-end reads were then merged using FLASH (V1.2.11).^26^ Quality filtering was performed with fastp (Version 0.23.1), ensuring an average quality score of 30.^27^ High-quality reads were then compared against the Silva reference database for 16S rRNA^28,29^ and the UNITE database for ITS2^30^ using the UCHIME algorithm to detect and remove chimeric sequences.^31^ The resulting fasta files contained more than 10 000 reads per library. All reads were merged and imported into QIIME2 (v2023.5)^32^ and processed with Deblur using default parameters,^33^ trimming all sequences to 300 bp. Diversity indices were calculated in QIIME2 using core_diversity_analyses.py with default parameters, rarefying to 10 000 sequences. Alpha diversity was assessed using the Shannon index, while beta diversity (between samples) was determined using the phylogenetic-based weighted UniFrac metric.^34^

### EPFR measurement

Soil samples were analyzed to observe the presence of EPFRs using electron magnetic resonance (EPR). EPR measurements were performed using a Bruker EMX-20/2.7 EPR spectrometer (X-band) with dual cavities set to the following parameters: microwave frequency of 9.781 GHz, power of 0.641 mW, 3 scans, modulation amplitude of 4.000 G, modulation frequency of 100.000 kHz, center field of 3479.060 G, sweep width of 100.000 G, time constant of 0.640 ms, conversion time of 20.480 ms, sweep time of 41.943 s, resolution of 2048 points, and receiver gain of 6.32 × 10^3^. All measurements were conducted at room temperature. Quantitative analyses and *g*-factor value determinations were conducted using a fitting macro inside the IGOR PRO data processing software. 2,2-Diphenyl-1-picrylhydrazyl (DPPH) was used as a quantification and calibration standard.

### Laboratory-generated EPFR

Batches of EPFR-containing nanoparticles generated with 2-monochlorphenol (MCP) were produced and provided by the LSU Superfund Center Materials Core as previously described.^9^ The EPFR concentration was quantified by EPR and ranged from 10^16^ to 10^17^ spins per g. The measured *g*-factor values ranged from 2.0036 to 2.0044.^3,35^ These lab-generated EPFRs were selected for their similarity to field-collected samples, which exhibited comparable concentrations (∼10^16^ spins per g) and *g*-factor values between 2.0034 and 2.0040,^36,37^ making them suitable analogs for modeling microbial responses to predominantly oxygen centered EPFR exposure.

### Quantify viable bacteria and fungi

EPFR-containing nanoparticles were prepared to a final concentration from 10, 100, 1000 µg mL^−1^ in 1X PBS buffer and mixed with 1 mg soil from a sampling site (13) near the facility. The exposure duration was limited to 3 hours to capture short-term microbial responses while minimizing extensive radical decay and secondary transformation processes that can occur in complex soil matrices. The persistence of EPFRs in various aqueous solutions was assessed previously, showing half-lives ranging from 1.55 hours to 3.43 hours. Notably, EPFRs generated from 2-MCP and suspended in saline were still detectable a week later after drying the solution and examination of it by EPR.^38^ CuO/SiO_2_ nanoparticles without free radicals were used as controls. The soil and EPFR mixtures were incubated in a rotator for 3 hours at room temperature and then underwent serial dilution using 1X PBS buffer. For each dilution, 100 µL was spread with a sterile spreader onto the Luria–Bertani (LB) agar plates, containing 10 g of tryptone, 5 g of yeast extract, 10 g of NaCl, and 15 g of agar in 1 L of distilled water, adjusted to pH 7.5. The plates were incubated at 30 °C for 24 hours. After incubation, plates with 50–300 bacterial colonies and 5–50 fungal colonies were selected for counting. Colonies were enumerated, and the colony-forming units per gram of soil (CFU g^−1^) were calculated using the formula:CFU g^−1^ = Number of colonies × volume plated ratio × serial dilution factor/soil weight in gram

### Bacterial isolation and growth assay

Soil samples collected from the sites were subjected to serial dilution before being plated on LB agar plates. Pure bacterial colonies were isolated through streaking to cultivate bacterial strains. Identification of bacterial strains was done by sequencing the full-length 16S rRNA gene using primers 27f and 1492r. EPFR-containing and CuO/SiO_2_ nanoparticles were prepared to a final concentration at 100 µg mL^−1^. For each condition, 100 µL aliquots were added to sterile 96-well plates, with three replicates per condition. Individual bacterial colonies were inoculated into LB broth and incubated overnight at 30 °C with shaking at 250 rpm. The bacterial cells were then harvested by centrifugation at 3000×*g* for 10 minutes, with the supernatant discarded. The pellets were fully resuspended through pipetting and transferred to a clear-bottom 96-well plate (Costar, Corning). Bacterial density was measured by optical density (OD) at 600 nm using a Multimode Microplate Reader (Biotek Cytation) and normalized to equal OD600 using sterile 1X PBS buffer for inoculation to assay plate. Bacterial growth was monitored by OD600 readings every hour for 24 hours at room temperature using a microplate reader. Growth curves were analyzed using AMiGA software^39^ to calculate the area under the curve (AUC), assessing total bacterial growth under each condition.

### Wind data analysis

Surface wind data obtained at the Alexandria International Airport (Weather Station 722487–13935) were retrieved from the National Oceanographic and Atmospheric Administration (NOAA) Integrated Surface Database (ISD) using the importNOAA function in worldmet in R (v. 4.5.0). The Alexandria, LA airport hosts a NOAA-operated weather station approximately 30 km southeast of the Colfax, LA soil collection sites. Data were averaged for the period January 1, 2000 – December 31, 2023. A wind rose was then produced for the average data on annual and seasonal bases using the windRose function in the openair package in R.

### Statistical analysis

Equality of variances between groups was assessed using an F-test prior to performing statistical comparisons. Student's *t*-test with equal variance was applied to compare EPFR measurements between near and distal sites. Two-way Analysis of Variance (ANOVA) was used to evaluate the interactions between seasonality and geographic location on bacterial and fungal abundance. Pearson and Spearman correlations were calculated to examine the relationship between bacterial abundance and EPFR levels. Statistical significance for student's *t*-test and correlation analyses was determined using Benjamini–Hochberg adjusted *p*-values to account for multiple comparisons, with a significance threshold set at <0.05. All statistical analyses were performed using R packages (stats) in RStudio (2023.06.1 + 524) with default parameters.

## Results and discussion

### Microbial abundance

**Fig. 2:**
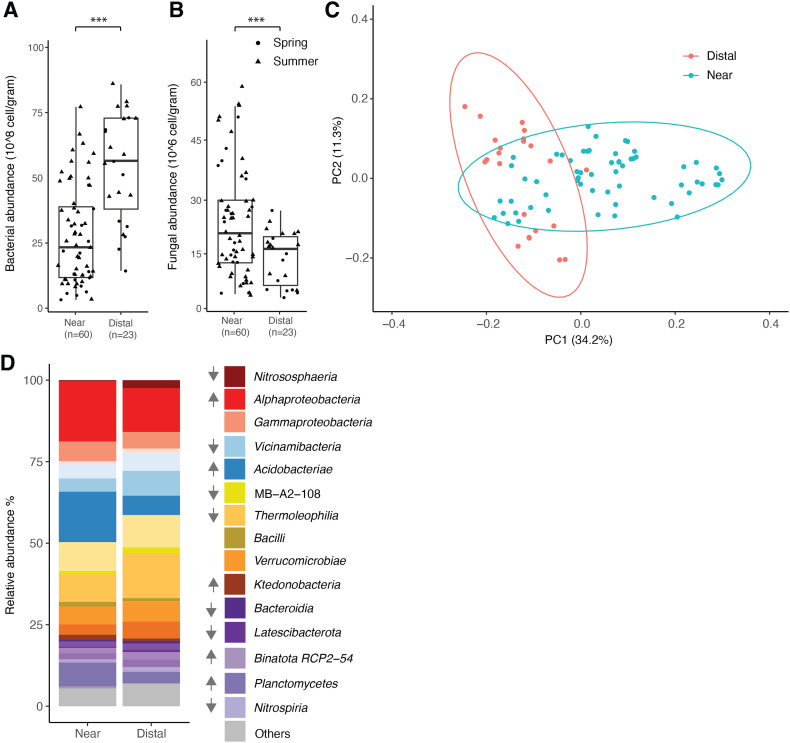
(A) and (B) Bacterial and fungal abundance in sites near and distal to the TT facility from spring (round) and summer (triangle) of Colfax, LA (*p* < 0.001). Asterisks indicate statistically significant differences between groups, with significance levels defined as *p < 0.05, **p < 0.01, and ***p < 0.001. (C) Principal coordinate analysis based on weighted UniFrac distance shows separated clustering bacterial composition in near and distal sites. Ellipses represent 95% confidence intervals around group centroids, calculated using stat_ellipse() in ggplot2. (D) Bacterial composition in near and distal sites. Relative bacterial abundance was presented here as the mean of samples. Upward arrows next to taxonomy indicate a significant increase (Benjamini–Hochberg adjusted *p* < 0.05) in relative abundance at nearby sites compared to distal sites, while downward arrows represent a significant decrease (adjusted *p* < 0.05) in relative abundance at nearby sites.

Microscope based counting found bacterial abundance near the TT facility at 31.6 ± 21.9 × 10^8^ cells per g (*n* = 60), around 50% lower than the distal sites at 62.3 ± 24.1 × 10^8^ cells per g (*n* = 23) [Fig. 2A]. In contrast, fungal abundance at the near sites was 22.7 ± 15.9 × 10^6^ cells per g (*n* = 60), nearly double compared to 12.5 ± 7.7 × 10^6^ cells per g at the distal sites (*n* = 23) [Fig. 2B]. A two-way ANOVA indicated a significant difference in bacterial abundance between near and distal sites (*F*_1,79_ = 34.555, *p* < 0.001) and a less pronounced but still significant variation across spring and summer (*F*_1,79_ = 11.076, *p* < 0.01). The interaction between site and season, however, was not significant (*F*_1,79_ = 0.147, *p* = 0.702), as shown in the roughly parallel lines in the interaction plot [Fig. S3A]. For fungal abundance, a significant difference was observed across sites (*F*_1,79_ = 8.477, *p* < 0.01), while neither seasonal variation (*F*_1,79_ = 0.336, *p* = 0.564) nor the interaction between site and season (*F*_1,79_ = 0.876, *p* = 0.352) showed significant difference.

### Microbial composition

The diversity of microbial communities was assessed using the 16S rRNA gene as a phylogenetic marker for bacteria and ITS2 gene for fungi. Analysis of the Shannon diversity index revealed no significant differences between sites near and distant from the facility, indicating that the presence of the TT facility does not directly affect the alpha diversity of soil bacterial communities [Fig. S3B]. To assess beta diversity, a Principal Coordinates Analysis (PCoA) was conducted, showing a clear separation between near and distant sites along PC1, which accounted for 34.2% of the variation in the microbiome [Fig. 2C]. Seasonal variations were also observed, with spring and summer samples separating along the PC2 axis, explaining 11.3% of the variation [Fig. S3C]. In contrast, soil depth had a minimal effect on bacterial communities, as the surface (0–1 cm) and deeper layers (5–10 cm) exhibited similar microbial profiles. This pattern aligns with previously reported high microbial community turnover rate in the topsoil, particularly in warm, moist, and biologically active environments.^40,41^ This trend is supported by beta diversity measurements indicated minimal within-site distances, with the most pronounced differences driven by proximity to the facility [Fig. S3D]. Comparative analysis of the relative abundance of bacterial taxa at the class level revealed notable differences between sites near and distal from the facility. Notably, five bacterial classes were significantly enriched near the facility (adjusted *p* < 0.05), including *Alpha-proteobacteria*, *Planctomycetes*, *Ktedonobacteria* (*Chloroflexi*), *Acidobacteriae*, and *Binatota* RCP2-54. Conversely, seven bacterial classes were depleted near the facility, including *Vicinamibacteria* (*Acidobacteriota*), *Bacteroidia* (*Bacteroidetes*), *Latescibacterota*, MB-A2-108, *Thermoleophilia* (*Actinobacteria*), *Nitrospiria* (*Nitrospirae*), and archaeal taxa *Nitrososphaeria* (*Crenarchaetoa*) [Fig. 2D]. In contrast to bacterial composition, there is no clear separation for fungal community between near and distal sites at the class level [Fig. S4A], both sites are dominated by *Agaricomycetes* (40.3%) in the phylum *Basidiomycota*, followed by an unclassified class (11.6%) and *Eurotiomycetes* (10.1%) in the phylum *Ascomycota* [Fig. S4B]. No fungal class showed a significant change in abundance with an adjusted *p*-value of <0.05.

### EPFR concentration and characteristics

**Fig. 3:**
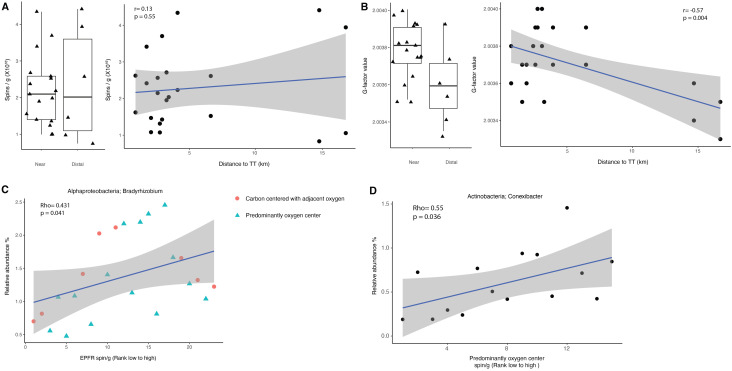
(A) Abundance of EPFR (*y*-axis) plotted against distance to the TT facility (*x*-axis). (B) *G*-factor values of EPFR (*y*-axis) plotted against distance to the TT facility (*x*-axis). (C) Spearman correlations between the relative abundance of *Bradyrhizobium* (*Alpha-proteobacteria*), with EPFR abundance: carbon-centered radicals with adjacent oxygen (red circles) and predominantly oxygen-centered radicals (green triangles). (D) Spearman correlations between the relative abundance of *Conexibacter* (*Actinobacteria*) with EPFR abundance for predominantly oxygen-centered radicals. The Benjamini–Hochberg adjusted *p*-value was used to assess the significance of correlations.

Physicochemical changes in the soil environment near the TT facility may contribute to the shifts in microbial communities. Given that EPFRs have been detected in air samples at the study site,^22^ one plausible driver is oxidative stress resulting from chronic exposure to EPFRs emitted by the facility. The biological impact of EPFRs varies depending on both their concentration and the nature of their paramagnetic centers, therefore, both parameters were quantified in the corresponding soil samples. The average abundance and standard deviation of EPFR in the near sites is 2.23 ± 0.93 × 10^16^ spins per g, compared to 2.38 ± 0.15 × 10^16^ spins per g in the distal sites, with no significant difference (*p* = 0.77) and no clear trend related to distance (*r* = 0.13, *p* = 0.55) [Fig. 3A]. Given the study site is not classified as a Superfund site, these EPFR concentrations are lower than those reported at Superfund sites, where soil EPFR levels reach around 10^17^ spins per g.^1,2,15,42–46^ The similar EPFRs concentrations between near and distal sites may be attributed to elevated microbial activity near the facility mitigating EPFR concentrations, thereby establishing a new equilibrium. Another possibility is that tree coverage near the facility blocks particulate deposition, allowing particulates to rise and disperse farther from the site, consistent with observations of similar EPFR concentrations in air samples at both near and distal sites.^22^ While the TT facility represents a significant local source of combustion-derived EPFRs in the study area, background sources such as vehicular emissions, residential and agricultural combustion, and naturally occurring radical formation in soils may also contribute to the regional radical load. These additional sources may partially explain the relatively uniform EPFR concentrations observed across sampling locations. Further investigation, including vegetation mapping and sampling extending beyond a 17-km radius, may help identify areas with lower airborne EPFR exposure and further explore these dynamics.

The paramagnetic center of free radicals influences oxidative reactivity, characterized by the *g*-factor. The soil samples exhibited *g*-factor values ranging from 2.0033 to 2.0040, suggesting that the EFPRs in the soil were primarily carbon-centered with adjacent oxygen atoms or predominantly oxygen centered.^47,48^ Interestingly, *g*-factor values in the near sites are significantly difference from the distal sites (*p* = 0.02), showing a trend of decreasing *g*-factor values with increasing distance from TT (*r* = −0.57, *p* = 0.004) [Fig. 3B], indicating unpaired electrons in a more electronegative and reactive environment at near sites. This spatial variation suggests a greater contribution of oxygen-centered radicals relative to carbon-centered radicals near the facility, indicating increased oxidative reactivity. Such radicals have been linked with adverse health effects in humans and may exert stronger effects on microbial community responses at sites closer to the facility.^49^ In contrast, air samples showed similar *g*-factor values across near and distal sites,^22^ implying that chemical and biological processes unique to the soil environment may drive the transformation of EPFRs after deposition.

### Bacterial taxa correlate with EPFRs

Given the geographic variation in EPFR composition from *g*-factor values, site-specific differences in both EPFR and bacterial taxa abundance may help identify bacterial groups that are responsive to EPFR exposure. Therefore, correlation analyses were conducted to assess relationships between bacterial taxa at the genus level and EPFR concentrations. Pearson correlation analysis did not reveal significant correlations (*p*-value < 0.05) between EPFR abundance and the bacterial taxa at genus level. However, Spearman correlation analysis identified 27 bacterial genus with moderate positive or negative correlations with EPFR abundance. Notably, *Bradyrhizobium* in *Alpha-proteobacteria* (Rho = 0.431) [Fig. 3C] and *Mycobacterium* in *Actinobacteria* (Rho = 0.493) [Fig. S5A] showed positive correlations with EPFR concentration, while *Anaerolineaceae* in the phylum *Chloroflexi* (Rho = −0.402) showed a negative correlation with EPFR concentration [Fig. S5B].

To further explore the influence of EPFR paramagnetic centers on the soil microbiome, separate correlation analyses were performed for samples with *g*-factor values below 2.0037, classified as carbon-centered radicals with adjacent oxygen, and those with *g*-factor values above 2.0037 as predominantly oxygen-centered radicals. Among the samples categorized as carbon-centered radicals with adjacent oxygen, 15 bacterial taxa exhibited significant correlations with EPFR abundance. In contrast, for samples dominated by oxygen-centered radicals, 35 bacterial genera were found to correlate with EPFR abundance. Specifically, *Conexibacter* (Rho = 0.55) [Fig. 3D] and *Gaiellales* (Rho = 0.5) [Fig. S5C] in *Actinobacteria* showed positive correlations with EPFR abundance, while an uncultured *Armatimonadota* (Rho = −0.76) exhibited a negative correlation [Fig. S5D]. Interestingly, the correlations were stronger for bacterial taxa associated with EPFRs predominantly oxygen-centered compared to EPFRs containing carbon-centered radicals with adjacent oxygen. This suggests that the more reactive oxygen-centered EPFRs may have a stronger influence on local microbial community structure, potentially driving the observed spatial variation in microbial composition. These positive correlations may reflect the adaptation of certain microbes to heightened oxidative stress, such as *Rhizobium* in host-associated environments^50^ and *Actinobacteria*, which are known for producing secondary metabolites with antioxidant properties.^51^

### Microbial responses to laboratory generated EPFR

**Fig. 4:**
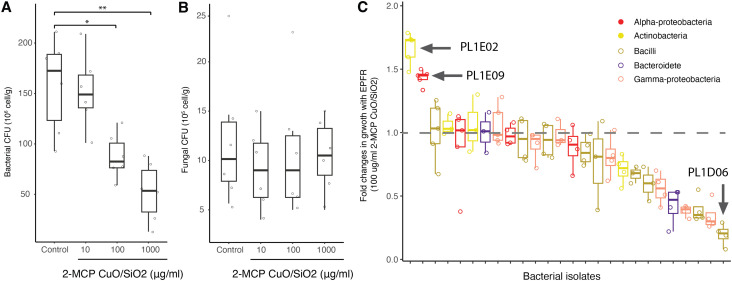
(A) and (B) Box plot of viable counts of bacteria and fungi (CFU) following a 3 h exposure to increasing concentrations of EPFRs (**p ≤ 0.01, *p ≤ 0.05). (C) Box plot of fold changes in bacterial growth (AUC) with EPFR exposure compared to without EPFR, with the dashed grey line representing equal growth under both conditions. Bar colors correspond to taxonomic classification and follow the same color scheme used in Fig. 2 for soil microbiome composition. Results are based on two independent trials.

Environmental oxidants can penetrate microbial cells and disrupt the balance of intracellular antioxidant defenses. The cellular response to ROS is highly regulated, and elevated levels of external free radicals can overwhelm these systems, leading to oxidative damage to cellular components such as DNA and impairing growth. Previous studies have shown diverse microbial responses to oxidative stress, including changes in growth patterns, membrane composition, and antioxidant production.^19^ Laboratory-generated EPFRs provide a controlled approach to directly assess the impact of oxidative stress on soil microbiome. In our microcosm experiment, a 3-hour exposure to oxygen-centered EPFRs reduced bacterial CFU in a dose-dependent manner, starting at 100 µg mL^−1^ [Fig. 4A]. This exposure level corresponds to a free radical concentration of approximately 10^16^ spins per g, consistent with the EPFR concentrations observed in the field. In contrast, fungal CFU counts remained unchanged even at the highest EPFR concentration of 1000 µg mL^−1^ [Fig. 4B], which is approximately double the maximum EPFR concentration detected in the soil. These findings suggest that bacteria are generally more sensitive to EPFR exposure, while fungi exhibit greater resistance, even at high concentrations. Although total EPFR concentrations were relatively uniform across sampling locations, the chemical characteristics of the radicals differed spatially. In particular, higher *g*-factor values observed at sites closer to the TT facility suggest a greater contribution of oxygen-centered radicals compared with more distal locations. In our microcosm experiment, exposure to oxygen-centered radicals produced differential responses between bacterial and fungal communities. Together, these results support the hypothesis that EPFR characteristics along with concentration, may contribute to shaping microbial communities in soils near the facility.

The relatively stable fungal community composition and increased fungal abundance near the facility may be attributed to fungi's inherent resistance to oxidative stress^52,53^ or ecological niche expansion resulting from reduced bacterial abundance. Several factors may explain fungi's greater resilience. First, most bacteria have a peptidoglycan-based cell wall with relatively limited structural complexity, making them more susceptible to oxidative damage.^19^ In contrast, fungi possess more robust cell walls made of polysaccharides, chitin, and glycoproteins, offering better physical protection against oxidative stress.^54^ Second, fungi's eukaryotic cellular structure includes compartmentalized organelles such as mitochondria and peroxisomes, which contribute to more sophisticated stress response mechanisms.^52,53^ Third, fungi employ oxidative metabolic pathways for essential biological functions. For example, fungal laccases contribute to detoxification processes and catalyze the oxidation of aromatic substrates for melanin synthesis, a cell wall pigment that protects fungi against UV radiation.^55,56^ These adaptations may influence fungi's ecological roles and responses to environmental pollutants such as EPFRs.

Bacterial growth curves have been commonly used to assess bacterial physiologies under microbial stressors.^57^ To investigate specific bacterial responses to EPFRs, a selective panel of 26 bacterial isolates from Colfax soil was tested for growth under EPFR exposure at 100 µg mL^−1^, a concentration previously shown to suppress bacterial CFU counts [Fig. 4A]. These isolates represent major taxa found in the soil, including *Alpha-proteobacteria* (4), *Gamma-proteobacteria* (7), *Bacteroidetes* (2), *Bacilli* (9), and *Actinobacteria* (4). However, other major taxa like *Acidobacteria* and *Chloroflexi* have no cultivated representatives yet due to the challenges of growing these groups under standard laboratory conditions.^58,59^

The results revealed a wide range of growth responses to EPFR exposure, supporting the hypothesis that bacterial composition changes are driven by taxa better adapted to oxidative stress. Most strains exhibited either comparable or reduced growth in the presence of EPFRs compared to control nanoparticles [Fig. 4C], as demonstrated by the growth curve of *Paenibacillus taihuensis* PL1D06 [Fig. S6A]. However, two isolates, *Rhizobium zeae* PL1E09 from *Alpha-proteobacteria* and *Gordonia hongkongensis* PL1E02 from *Actinobacteria*, showed enhanced growth under EPFR exposure [Fig. S6B and C]. Interestingly, both isolates belong to taxa that positively correlated with EPFR abundance, supporting the hypothesis that changes in bacterial composition are driven by bacterial taxa better adapted to oxidative stress.^19^ The ability to exhibit faster growth and higher carrying capacity in response to EPFR exposure suggests that these microbes may possess unique metabolic capabilities, enabling them to degrade aromatic compounds or synthesize antioxidant products, making them valuable genomic resources for understanding how EPFR toxicity is mitigated and bioremediation applications. Future studies incorporating metagenomic sequencing of samples from near and distal sites would be valuable to elucidate the changes in microbial functional pathways across bacterial and fungal taxa. Complementary approaches, such as enzymatic assays for microbial ROS-scavenging activity or gene expression analysis of oxidative stress markers like *katG* (catalase) or *sodA/B* (superoxide dismutase),^60^ could provide direct evidence of microbial oxidative stress responses. Additionally, in natural soil matrices, free radicals may undergo partial quenching or transformation due to interactions with organic matter, minerals, and other reactive components. As a result, the effective radical exposure experienced by microorganisms may differ from the nominal dose applied. Future studies could investigate radicals with different chemical characteristics, such as carbon-centered radicals with *g*-factor values around 2.0028, to examine how variations in radical type influence microbial responses. Controlled dosing of laboratory-generated EPFRs in soil microcosm longitudinal experiments would also allow quantification of transformation rates of EPFRs and profile microbial community changes in soil over time,^18^ potentially providing a more comprehensive understanding of microbial adaptations to EPFR exposure.

### Environmental implications

Oxidative stress, which can be induced by EPFR exposure, has been shown to drive bacterial mutations and subsequent development of antibiotic resistance,^61^ these disruptions to the microbiome could carry significant public health risks. Soil microbiome act as reservoirs of microbial populations shared among plants, animals, and humans through direct or indirect exposure, such as farming and gardening.^62^ These microbial populations include beneficial members which promote plant growth and pest resistance, alongside harmful pathogens that contribute to zoonotic disease transmission and the spread of antimicrobial resistance.^62,63^ Moreover, the observed increase in fungal abundance in the affected soil may elevate the risk of respiratory illnesses,^64^ especially among residents with compromised health due to EPFR exposure.^23^

Beyond health risks, shifts in soil microbiomes near the TT facility can affect key microbial functions related to local nutrient cycling.^65^ For example, the enrichment of the genus *Rhizobium* within *Alpha-proteobacteria*, known for their nitrogen-fixing capabilities,^66^ alongside the decline in ammonia-oxidizing archaea *Nitrososphaeria*^67,68^ and bacteria *Nitrospiriae*^67,69,70^ near the facility may suggest potential nitrogen limitation. A similar decline in *Nitrospiriae* has been observed in experiments involving biochar-associated EPFR amendment.^18^ Furthermore, altered abundance of multiple *Acidobacteria* class near the facility may indicate changes in soil pH.^20^ These findings highlight the need for expanded field sampling combined with thorough chemical analyses, including soil pH, nutrient levels, and EPFR concentrations, to better understand the chemical drivers of microbial community changes.

Taken together, EPFRs have the potential to drive changes in soil microbiome, alongside other environmental contaminants and stressors, such as heavy metals and pesticides.^71,72^ Our findings underscore the need for further research, policy development, and mitigation efforts to address EPFR-related impacts on both ecological systems and human health.

## Conflicts of interest

There are no conflicts to declare.

## Supplementary Material

EM-028-D5EM00439J-s001

## Data Availability

Original data have been deposited to NCBI Sequence Read Archive under Bioproject PRJNA1212207. The accession numbers for sequences of microbiome composition and transcriptome reported in this paper are Sequence Read Archive: SAMN46387143-46387283. Supplementary information (SI): additional data supporting site characterization, environmental context, microbial community analyses, and experimental validation. Fig. S1 presents historical air emissions data (2003–2024) summarizing annual net explosive waste treated at the facility. Fig. S2 provides long-term (2000–2023) annual and seasonal wind patterns from a nearby NOAA weather station. Fig. S3–S4 include additional analyses of bacterial and fungal community structure, diversity, and beta diversity comparisons between sites and seasons. Fig. S5 shows correlations between selected bacterial taxa and EPFR abundance for different radical types. Fig. S6 presents growth responses of representative bacterial strains to laboratory-generated EPFR exposure. See DOI: https://doi.org/10.1039/d5em00439j.
