# Supplementary material for: Shifts in soil microbiome surrounding a thermal treatment facility for hazardous waste: the hidden impact of environmentally persistent free radicals
Source: Environ Sci Process Impacts. 2026 Apr 27;28(5):1479–90. doi: 10.1039/d5em00439j (PMC13113242; doi:10.1039/d5em00439j)
Supplement: EM-028-D5EM00439J-s001 [file EM-028-D5EM00439J-s001.pdf]

Supplementary Information (SI) for Environmental Science: Processes & Impacts.
This journal is © The Royal Society of Chemistry 2026

Supplemental figures

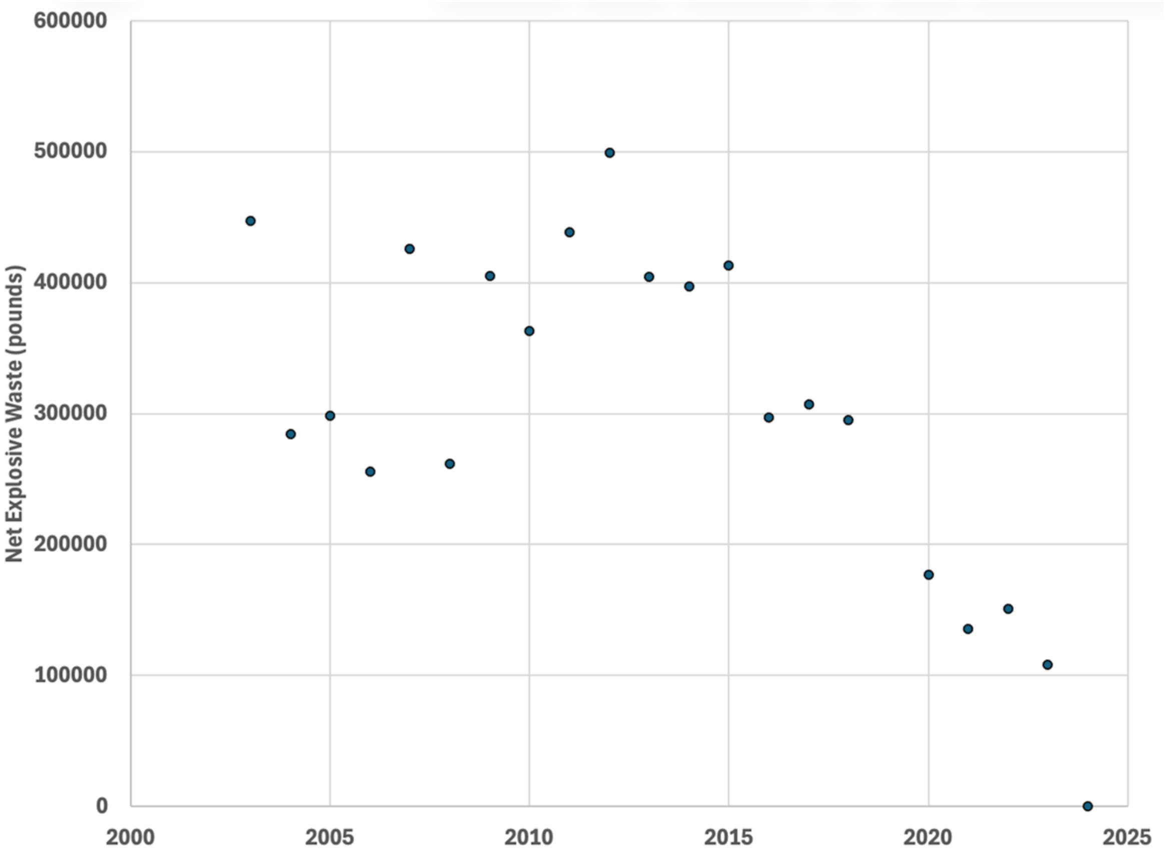

Figure S1. Annual net explosive waste treated at the thermal treatment facility from 2003–2024 based on publicly available air emissions reports retrieved from the Louisiana Department of Environmental Quality (LDEQ) Electronic Document Management System (EDMS). The data represent annual totals reported in facility air emissions reports and reflect the net quantity of explosive waste treated through thermal operations each year.

Figure S2. Annual average and seasonal average wind roses from the NOAA Weather Station (722487-13935) at the Alexandria International Airport, Alexandria, LA. Data were obtained for the time period between January 1, 2000 and December 31, 2023.

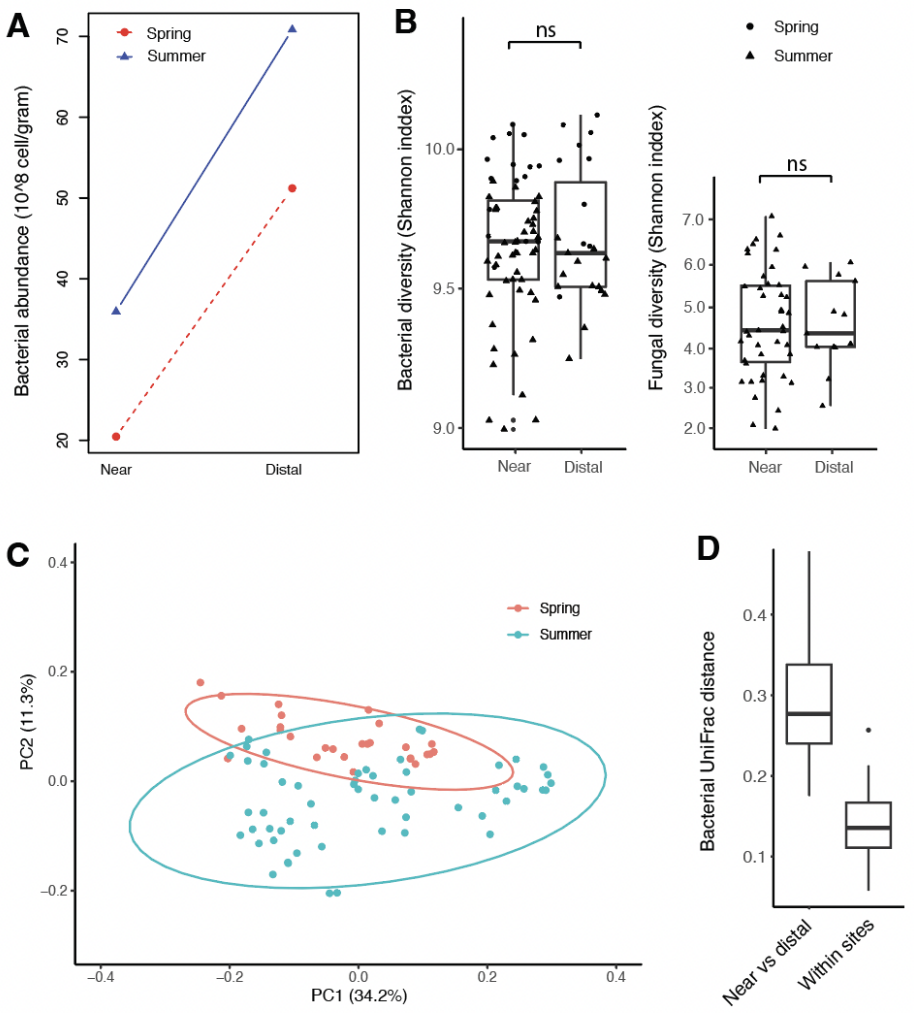

Figure S3 A. Interaction plot of bacterial abundance near and distal to the facility between spring and summer. **B.** Bacterial and fungal diversity are similar between near and distal sites as assessed by Shannon alpha diversity indices. **C.** Principal coordinate analysis based on weighted UniFrac distance shows separated clustering bacterial composition in spring and summer samples along the PC2 axis. **D.** Box-whisker plots of the weighted UniFrac distances exhibit greater difference in bacterial composition between near and distal sites compared to within sites.

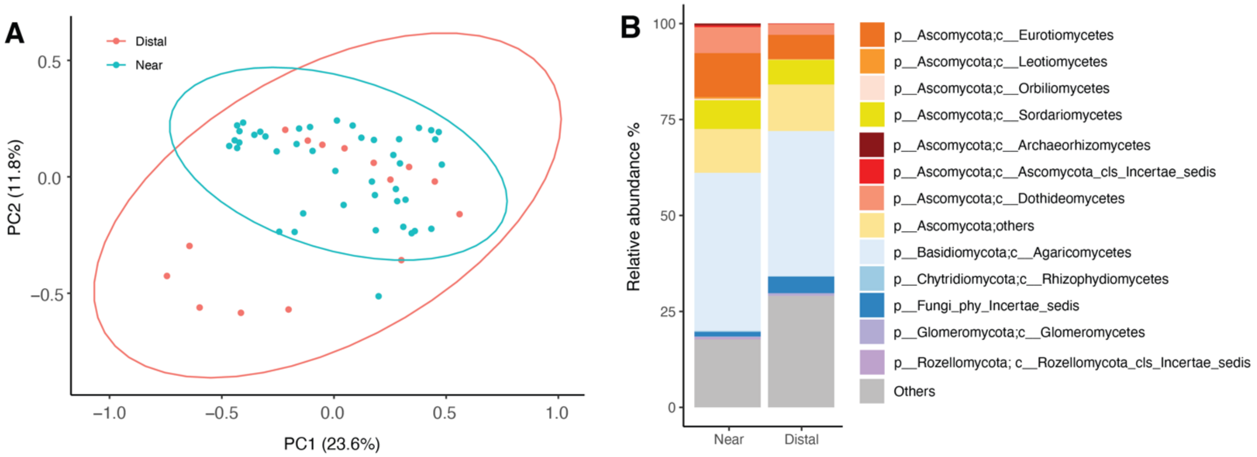

Figure S4 A. Principal coordinate analysis shows similar fungal composition in near and distal sites. **B.** Fungal composition in near and distal sites. Relative fungal abundance was presented here as the mean of samples.

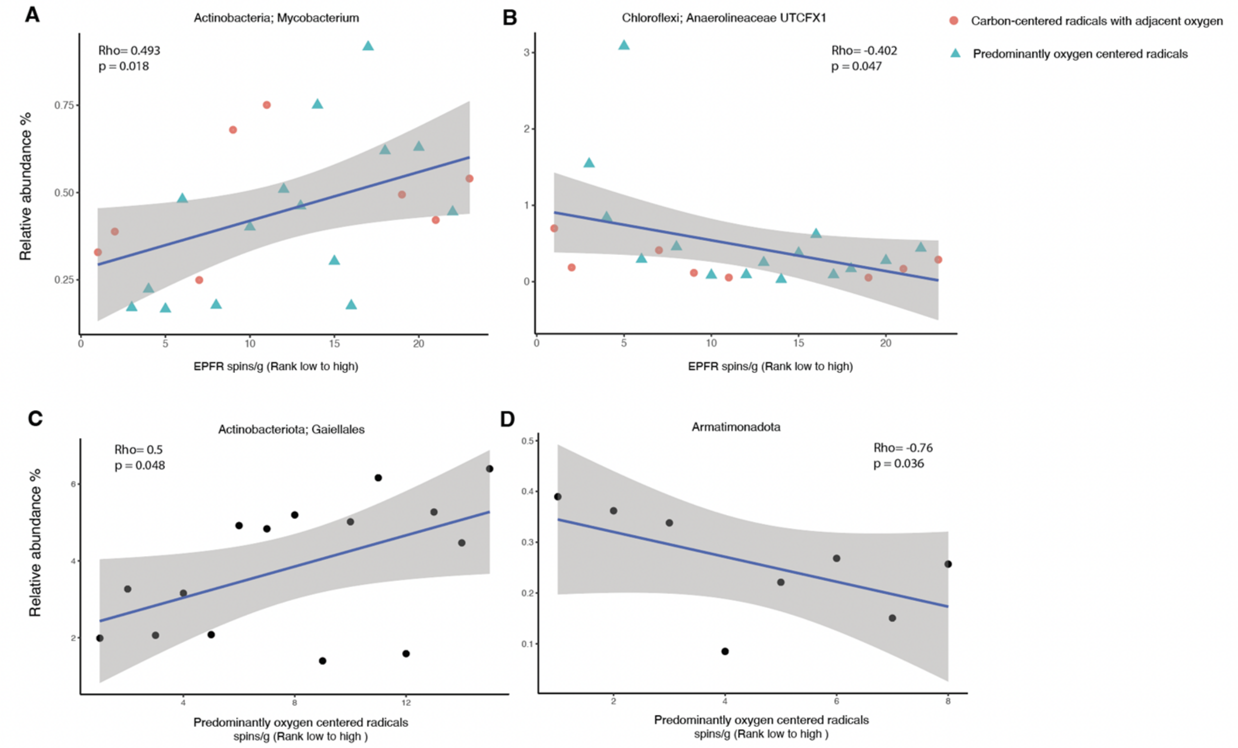

Figure S5 A-B. Spearman correlations between the relative abundance of *Mycobacterium* (*Actinobacteria*), and *Anaerolineaceae* (*Chloroflexi*) with EPFR abundance: carbon-centered

radicals with adjacent oxygen (red circles) and predominantly oxygen-centered radicals (green triangles). **C-D**. Spearman correlations between the relative abundance of *Gaiellales* (*Actinobacteria*), and an uncultured *Armatimonadota* species with EPFR abundance for predominantly oxygen-centered radicals. Grey shading indicates the 95% confidence interval around the estimated mean, calculated using geom_smooth() in ggplot2 with a linear model (method = "lm")

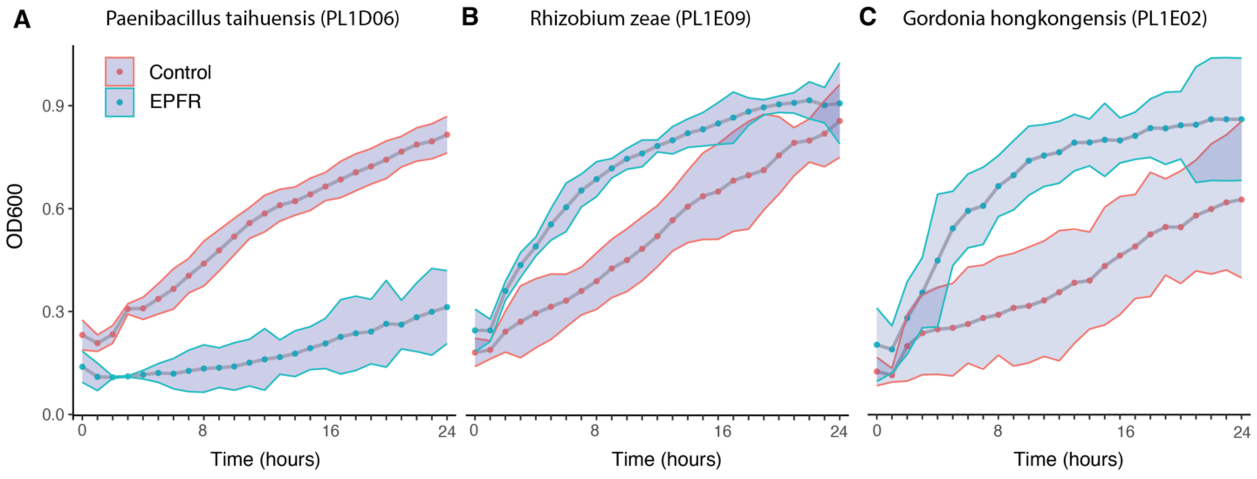

Figure S6 Growth curves of bacterial strains *P. taihuensis* PL1D06 **(A)**, *R. zeae* PL1E09 **(B)**, and *G. hongkongensis* PL1E02 **(C)** with (green dots) or without laboratory-generated EPFR (red dots). Bold lines plot the mean of OD600, and bands indicate the predicted 95% confidence interval based on biological replicates.
